# The burden of Parkinson’s disease in the Middle East and North Africa region, 1990–2019: results from the global burden of disease study 2019

**DOI:** 10.1186/s12889-023-15018-x

**Published:** 2023-01-16

**Authors:** Saeid Safiri, Maryam Noori, Seyed Aria Nejadghaderi, Seyed Ehsan Mousavi, Mark J. M. Sullman, Mostafa Araj-Khodaei, Kuljit Singh, Ali-Asghar Kolahi, Kurosh Gharagozli

**Affiliations:** 1 Neurosciences Research Center, Aging Research Institute, Tabriz University of Medical Sciences, Tabriz, Iran; 2 Research Center for Integrative Medicine in Aging, Aging Research Institute, Tabriz University of Medical Sciences, Tabriz, Iran; 3 Department of Community Medicine, Faculty of Medicine, Tabriz University of Medical Sciences, Tabriz, Iran; 4 Student Research Committee, School of Medicine, Iran University of Medical Sciences, Tehran, Iran; 5 School of Medicine, Shahid Beheshti University of Medical Sciences, Tehran, Iran; 6 Systematic Review and Meta-Analysis Expert Group (SRMEG), Universal Scientific Education and Research Network (USERN), Tehran, Iran; 7 Student Research Committee, Tabriz University of Medical Sciences, Tabriz, Iran; 8 Department of Life and Health Sciences, University of Nicosia, Nicosia, Cyprus; 9 Department of Social Sciences, University of Nicosia, Nicosia, Cyprus; 10 Department of Medicine, Griffith University, Southport, QLD Australia; 11 Social Determinants of Health Research Center, Shahid Beheshti University of Medical Sciences, Tehran, Iran; 12 Brain Mapping Research Center, Shahid Beheshti University of Medical Sciences, Tehran, Iran

**Keywords:** Parkinson’s disease, Middle East and North Africa, Epidemiology, Mortality, Disability-adjusted life-years

## Abstract

**Background:**

Parkinson’s disease (PD) remains a common disabling progressive neurodegenerative disorder. We aimed to report the prevalence, death and disability-adjusted life-years (DALYs) attributable to PD in the Middle East and North Africa (MENA) region and its 21 countries by age, sex and socio-demographic index (SDI), between 1990 and 2019.

**Methods:**

Publicly available data on the burden of PD in the MENA countries were retrieved from the Global Burden of Disease (GBD) 2019 project. The results are presented with age-standardised numbers and rates per 100,000 population, along with their corresponding 95% uncertainty intervals (UIs).

**Results:**

In 2019, PD had an age-standardised point prevalence of 82.6 per 100,000 population in MENA and an age-standardised death rate of 5.3, which have increased from 1990 to 2019 by 15.4% and 2.3%, respectively. In 2019, the age-standardised DALY rate of PD was 84.4, which was 0.9% higher than in 1990. The highest and lowest age-standardised DALY rates of PD in 2019 were found in Qatar and Kuwait, respectively. Also in 2019, the highest number of prevalent cases and number of DALYs were found in the 75–79 age group for both sexes. In 2019, females in MENA had an overall higher DALY rate. Furthermore, from 1990 to 2019 the burden of PD generally decreased with increasing socio-economic development, up to an SDI of around 0.4, and then increased with higher levels of SDI.

**Conclusion:**

An upward trend was observed in the point prevalence of PD over the last three decades. This highlights the need to allocate more resources for research. Furthermore, properly equipped healthcare services are needed for the increasing number of patients with PD.

**Supplementary Information:**

The online version contains supplementary material available at 10.1186/s12889-023-15018-x.

## Introduction

Parkinson’s disease (PD) is the second most common neurodegenerative disorder, and is characterised by decreased levels of dopamine, due to the loss of dopaminergic neurons in the substansia nigra [1]. At disease onset, motor symptoms of parkinsonism (e.g., tremors, dyskinesia and rigidity) are common, whereas other non-motor symptoms develop (e.g., constipation, anosmia and cognitive dysfunction) as the disease progresses [1]. Exposure to methamphetamines, dairy products and pesticides, or via comorbidities (e.g., cancer and traumatic brain injury) predispose individuals to developing PD through mechanisms such as mitochondrial dysfunction, oxidative stress and neuroinflammation [2, 3]. The prognosis differs according to the subtype of PD, but in general a life expectancy of 7 to 14 years is noted following the diagnosis [4].

The Middle East and North Africa (MENA) region consists of 21 countries with wide ranging cultural and lifestyle-related differences. Nevertheless, some region-specific environmental, cultural, genetic and health-system related factors make MENA an important region in which to evaluate the epidemiology and natural history of PD [5]. In the Eastern Mediterranean Region (EMR), the age-standardised prevalence rate of PD increased by 42.3% over the period 1990–2016, and reached 87.1 per 100,000 in 2016 [6]. Furthermore, globally there was a 155.5% increase in the number of prevalent cases of PD between 1990 and 2019 [7]. Moreover, the PD-attributable disability-adjusted life year (DALY) rates increased significantly with increases in the socio-demographic index (SDI) [6]. In addition, PD was found to be more common among males and its prevalence increased with advancing age, peaking in the 85–89 and 90–94 age groups in males and females, respectively [6].

Although the global burden of PD has been previously reported using data from the Global Burden of Disease (GBD) study 2016 [8] and GBD study 2019 [9], the regional-specific patterns are not usually considered in the global-level papers, and these may differ substantially from global patterns. However, one study reported the burden of neurodegenerative diseases in the EMR between 1990 and 2016, but this study is now outdated and estimates were not provided for some MENA countries, such as Turkey [6]. Therefore, the present study reported the numbers and rates of PD in the MENA region, in terms of the prevalence, death and DALYs between 1990 and 2019, by location, age, sex and SDI.

## Methods

### Overview

The GBD study monitors the burden of diseases and injuries in 204 countries and territories [7]. There are 21 countries in the MENA region, including Afghanistan, Algeria, Bahrain, Egypt, Iran, Iraq, Jordan, Kuwait, Lebanon, Libya, Morocco, Oman, Palestine, Qatar, Saudi Arabia, Sudan, the Syrian Arab Republic, Tunisia, Turkey, the United Arab Emirates and Yemen. GBD 2019 has data from 1990 to 2019, but there have been a number of improvements in the utilised methodologies and modelling strategies since 2017, which are described in detail elsewhere [7, 10]. The data on the fatal and non-fatal estimates can be obtained via the following links https://vizhub.healthdata.org/gbd-compare/ and http://ghdx.healthdata.org/gbd-results-tool.

### Case definition and data sources

PD is a chronic and progressive neurological degenerative condition that results in the gradual loss of motor mobility and control. According to the GBD project, PD requires the presence of two or more of the following four symptoms: (1) tremors/trembling, (2) bradykinesia, (3) limb and torso stiffness, and (4) postural instability [7]. As there were significant differences between the prevalence data and the cause of death data, the mortality and morbidity estimates for PD were modelled together. Firstly, mortality data were collected from the vital registration systems of the individual countries, along with prevalence data from surveys and administrative data (e.g., claims data). Furthermore, a systematic review was conducted in GBD 2019 (September 2015 to August 2017) using the appropriate keywords [7]. Studies were not included if the samples were not representative of the population, had poor study designs, or conflicted with existing gold-standard data. Prevalent cases were obtained using claims data where a patient had one inpatient visit, two outpatient visits, or one outpatient and one inpatient visit, except for in the year 2000, as there were generally less claims than normal [7]. The data sources that were included in the modelling process can be found here: https://ghdx.healthdata.org/gbd-2019/data-input-sources.

### Disease model

The mortality rates were modelled by age, sex, and location using the cause of death ensemble model (CODEm) and the covariates included have been previously reported. All data on the incidence, prevalence, and mortality risk of PD were used to model PD prevalence by age, sex and location using a Bayesian meta-regression-based tool (DisMod-MR 2.1). In the model, remission was fixed at zero. In addition, studies that did not use the same case definition (i.e. two of the four main symptoms of PD) were cross-walked to the reference case definition. Additional details about the estimation process have been previously reported.

### Severity and years lived with disability

The three severity levels of PD (G20, G21, and G22) from the International Classification of Disease 10 (ICD-10) were used (see Table S1), along with the disability weights from the GBD Disability Weights Measurement Study [11]. Finally, the point prevalence of each severity category were multiplied by the severity-specific disability weights (DWs) in order to estimate the years lived with disability (YLDs).

### Compilation of results

The number of deaths in each age group were multiplied by the remaining life expectancy in that age group, as determined by the GBD standard life table, in order to model the years of life lost (YLLs). The YLDs and YLLs were added together to estimate the DALYs. All estimates were standardised and included 95% uncertainty intervals (UIs). Smoothing Splines models were used to investigate the relationship that SDI had with the burden of PD [12]. SDI is an indicator of socio-economic development using the mean income per capita, mean years of schooling for those aged 15 or older, and the total fertility rate for those under 25 years of age. The SDI ranges from least developed (0) to most developed (1). The analyses were all undertaken using R Software (V.3.5.2).

## Results

### The Middle East and North Africa region

In 2019, PD accounted for 309.9 thousand (95% UI: 265.0 to 362.8) prevalent cases in the MENA region. Also in 2019, the age-standardised point prevalence of PD was 82.6 (95% UI: 70.2 to 95.6) per 100,000 population, which shows a 15.4% (95% UI: 11.5 to 20.0) increase since 1990 (Table 1 and Table S2). PD was responsible for 16.8 thousand (95% UI: 14.6 to 21.6) deaths in 2019, with an age-standardised rate of 5.3 (95% UI: 4.6 to 6.9), an increase of 2.3% (95% UI: -10.0 to 19.0) since 1990 (Table 1 and Table S3). In 2019, the number of regional DALYs was 300.7 thousand (95% UI: 266.3 to 365.4), with an age-standardised rate of 84.4 (95% UI: 74.7 to 103.2) DALYs per 100,000 population, a 0.9% (95% UI: -10.2 to 15.7) increase since 1990 (Table 1 and Table S4).

**Table 1:** Prevalence, deaths, and DALYs for Parkinson’s disease in the Middle East and North Africa region for both sexes in 2019 and the percentage change in the age-standardised rates during the period 1990–2019. DALY = disability-adjusted-life-years. (Generated from data available from http://ghdx.healthdata.org/gbd-results-tool)

	**Prevalence (95% UI)**			**Death (95% UI)**			**DALY (95% UI)**		
	**Counts** **(2019)**	**ASRs** **(2019)**	**Pcs in ASRs** **1990–2019**	**Counts** **(2019)**	**ASRs** **(2019)**	**Pcs in ASRs** **1990–2019**	**Counts** **(2019)**	**ASRs** **(2019)**	**PCs in ASRs** **1990–2019**
**North Africa and Middle East**	**309,887** **(264,964, 362,774)**	**82.6** **(70.2, 95.6)**	**15.4** **(11.5, 20)**	**16,784** **(14,607, 21,649)**	**5.3** **(4.6, 6.9)**	**2.3** **(-10, 19)**	**300,698** **(266,277, 365,360)**	**84.4** **(74.7, 103.2)**	**0.9** **(-10.2, 15.7)**
**Afghanistan**	**7352** **(6145, 8807)**	**69.3** **(58.2, 82.4)**	**-3.3** **(-12.9, 8)**	**560** **(431, 715)**	**6.8** **(5.3, 8.5)**	**-2.3** **(-22.7, 20.3)**	**11,574** **(8845, 14,866)**	**113.8** **(88.7, 143.3)**	**-6** **(-25.7, 16.3)**
**Algeria**	**23,956** **(19,889, 28,839)**	**80.4** **(66.5, 96.8)**	**10.2** **(-1.1, 22)**	**1283** **(1019, 1583)**	**5.4** **(4.4, 6.7)**	**-18.7** **(-36.3, 4.6)**	**21,708** **(17,579, 26,334)**	**78.9** **(64.1, 95.1)**	**-18.1** **(-35.7, 3.6)**
**Bahrain**	**674** **(552, 825)**	**94** **(75.9, 113.4)**	**12.5** **(-5.2, 33.3)**	**21** **(15, 27)**	**6** **(4.2, 7.4)**	**-7.7** **(-29.9, 17.9)**	**467** **(340, 573)**	**88.5** **(64.1, 106.6)**	**-13.5** **(-32.1, 7.5)**
**Egypt**	**43,837** **(36,133, 52,603)**	**84.4** **(69.1, 101)**	**17.3** **(3.6, 30.7)**	**2439** **(1842, 3675)**	**6** **(4.6, 9.3)**	**12.2** **(-10.6, 38.2)**	**48,343** **(37,833, 67,875)**	**98.6** **(77.1, 142.4)**	**11.7** **(-9.4, 36.5)**
**Iran (Islamic Republic of)**	**56,514** **(47,335, 66,920)**	**85.1** **(70.2, 101.3)**	**13.1** **(10.4, 15.8)**	**2848** **(2432, 3155)**	**4.8** **(4.1, 5.3)**	**6.7** **(-12.6, 28.4)**	**48,779** **(42,378, 53,932)**	**76.8** **(66.9, 84.9)**	**3.1** **(-12.9, 20.5)**
**Iraq**	**14,382** **(11,802, 17,136)**	**74.2** **(61.1, 87.9)**	**5.1** **(-4.3, 17.1)**	**842** **(684, 1150)**	**5.7** **(4.6, 7.4)**	**22.8** **(-3.6, 58)**	**14,973** **(12,204, 20,254)**	**85.6** **(71, 115.2)**	**17.5** **(-5.7, 47.1)**
**Jordan**	**4127** **(3383, 4884)**	**77** **(63.4, 91.5)**	**3** **(-11.5, 16.6)**	**192** **(160, 228)**	**4.9** **(4.1, 5.8)**	**-13.2** **(-30, 8.6)**	**3534** **(3007, 4138)**	**74.2** **(63, 86.7)**	**-14** **(-29.8, 5)**
**Kuwait**	**1581** **(1292, 1923)**	**71.4** **(59.1, 86.1)**	**-8.5** **(-23.9, 9.9)**	**58** **(47, 69)**	**3.5** **(2.8, 4.2)**	**-25.1** **(-36.3, -11.7)**	**1052** **(894, 1255)**	**56.1** **(47.2, 66)**	**-26.8** **(-36.5, -14.7)**
**Lebanon**	**4065** **(3476, 4757)**	**77.6** **(66.5, 90.9)**	**9.3** **(-3.1, 23.6)**	**226** **(180, 312)**	**4.4** **(3.5, 6)**	**-14.1** **(-37.2, 15.7)**	**3662** **(3016, 4825)**	**69.5** **(57.3, 91.6)**	**-14.2** **(-35.2, 11.2)**
**Libya**	**3830** **(3183, 4453)**	**84.1** **(69.3, 98.3)**	**13.5** **(2.3, 27.9)**	**203** **(150, 265)**	**5** **(3.7, 6.5)**	**9.9** **(-18.1, 50.5)**	**3712** **(2836, 4719)**	**84.8** **(65.3, 108.4)**	**10.9** **(-15.2, 45.8)**
**Morocco**	**20,470** **(17,155, 23,848)**	**73.7** **(61.9, 85.8)**	**20.7** **(6.3, 39)**	**1390** **(1122, 1614)**	**6** **(4.9, 7)**	**46.6** **(16.7, 85.9)**	**25,269** **(20,568, 29,607)**	**96.6** **(79.3, 111.9)**	**39.6** **(14.4, 73.3)**
**Oman**	**1362** **(1125, 1661)**	**112.5** **(92.9, 135.2)**	**28** **(14, 44.8)**	**60** **(37, 70)**	**9.1** **(4.9, 10.7)**	**44.7** **(6.3, 96)**	**1097** **(785, 1265)**	**125.3** **(79, 144.5)**	**30.3** **(-2.2, 70.4)**
**Palestine**	**1517** **(1259, 1792)**	**78.3** **(65.2, 92.4)**	**2.1** **(-8.8, 14.4)**	**92** **(61, 107)**	**6.2** **(4, 7.2)**	**-2.5** **(-26.2, 36.2)**	**1614** **(1142, 1855)**	**93.1** **(64.9, 106.8)**	**-4.9** **(-26.4, 29.1)**
**Qatar**	**750** **(594, 951)**	**119.3** **(98.3, 142.9)**	**20.2** **(6.9, 36.3)**	**19** **(13, 26)**	**11.1** **(6.5, 14.7)**	**35.3** **(1.2, 78.3)**	**488** **(360, 643)**	**148.6** **(96, 194.6)**	**14.8** **(-11.6, 49.8)**
**Saudi Arabia**	**13,291** **(11,019, 15,910)**	**107.6** **(90.4, 127.8)**	**27.7** **(13.1, 45)**	**562** **(414, 665)**	**7.3** **(5, 8.7)**	**0.2** **(-22.2, 47.7)**	**11,587** **(9409, 13,541)**	**111** **(83.8, 129.8)**	**1.6** **(-19.9, 43.5)**
**Sudan**	**11,401** **(9527, 13,651)**	**69.7** **(58.3, 83.4)**	**2.1** **(-9.9, 16.1)**	**710** **(557, 898)**	**5.2** **(4.1, 6.5)**	**0.1** **(-24.7, 36.8)**	**13,238** **(10,419, 16,768)**	**85.5** **(67.8, 107.5)**	**-4.3** **(-26.5, 26.1)**
**Syrian Arab Republic**	**8349** **(6932, 9929)**	**81.6** **(67.8, 97)**	**11.6** **(-1.3, 26.8)**	**434** **(286, 555)**	**5.7** **(3.7, 7.2)**	**21.1** **(-8.4, 61.8)**	**7787** **(5490, 9816)**	**83.9** **(58.4, 104.9)**	**13.7** **(-12.2, 49.7)**
**Tunisia**	**9312** **(7834, 10,877)**	**79.6** **(66.9, 93.2)**	**15.1** **(1.8, 30.4)**	**485** **(356, 646)**	**4.6** **(3.4, 6)**	**12** **(-16.4, 45.8)**	**8271** **(6418, 10,676)**	**72.7** **(56.2, 93.7)**	**11.1** **(-13.8, 41)**
**Turkey**	**72,458** **(59,364, 86,774)**	**86.9** **(71, 104.1)**	**22.3** **(10.9, 35.5)**	**3838** **(2916, 7139)**	**4.9** **(3.7, 9.1)**	**-6.8** **(-27, 16.1)**	**62,178** **(48,654, 105,236)**	**76.1** **(59.4, 129.7)**	**-8.2** **(-25.9, 11.9)**
**United Arab Emirates**	**3131** **(2506, 3880)**	**116** **(97.6, 138.8)**	**11.8** **(0.6, 23.6)**	**90** **(60, 123)**	**8** **(4.8, 10.4)**	**-12.8** **(-32.3, 23.5)**	**2896** **(2100, 3866)**	**130.5** **(87, 167.2)**	**-10.6** **(-29.6, 21.4)**
**Yemen**	**7214** **(5874, 8683)**	**63.6** **(51.9, 76.4)**	**9.7** **(-1.8, 24.7)**	**413** **(322, 540)**	**4.6** **(3.6, 6)**	**21.9** **(-3.2, 58.7)**	**8163** **(6383, 10,687)**	**76.8** **(60.8, 98.7)**	**15.5** **(-7.8, 49.7)**

*DALY* Disability-adjusted life-year, *ASRs* Age-standardised rates, *PCs* Percentage changes, *UI* Uncertainty interval

### National level

**Fig. 1:**
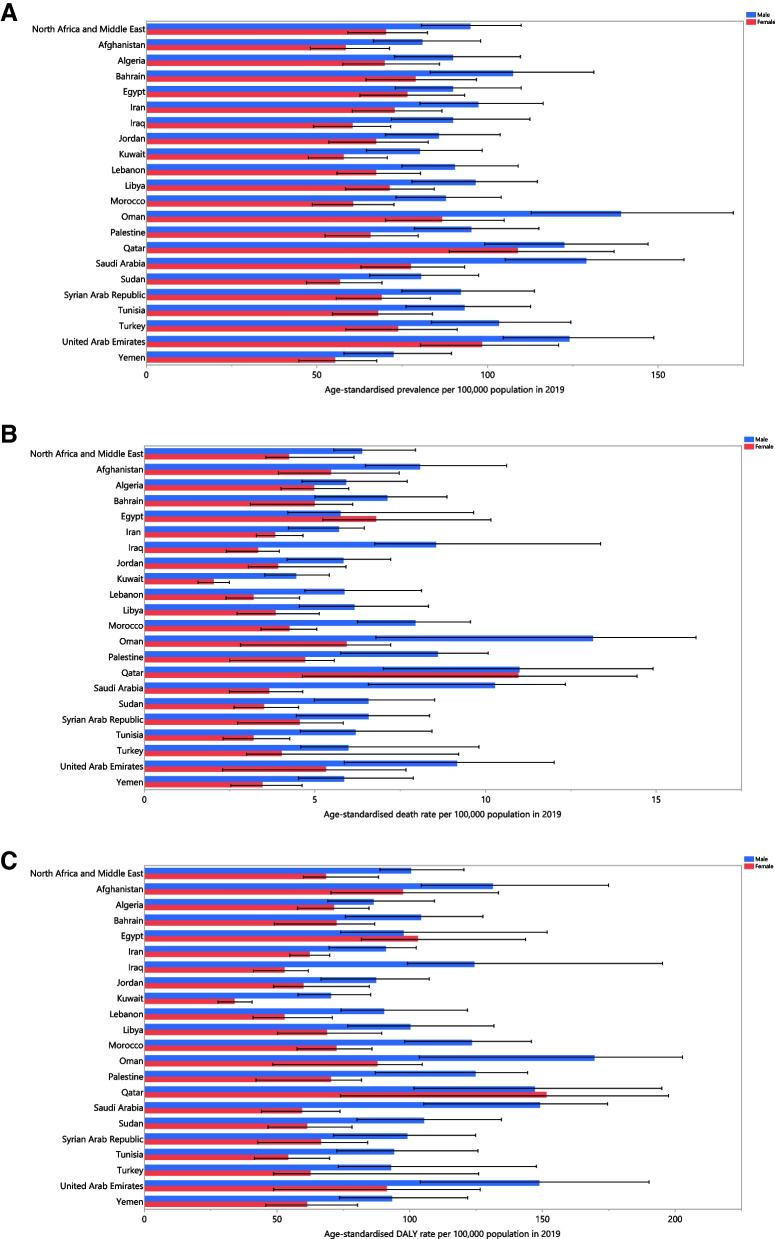
Age-standardised point prevalence (A), deaths (B), and DALYs (C) for Parkinson’s disease (per 100,000 population) in the Middle East and North Africa region in 2019, by sex and country. DALY = disability-adjusted-life-years. (Generated from data available from http://ghdx.healthdata.org/gbd-results-tool)

In 2019, the highest national age-standardised point prevalence of PD in MENA was 119.3, while the lowest was 63.6 cases per 100,000 population. The three countries with the highest estimated age-standardised point prevalence of PD in 2019 were Qatar [119.3 (95% UI: 98.3 to 142.9)], the United Arab Emirates [116.0 (95% UI: 97.6 to 138.8)] and Oman [112.5 (95% UI: 92.9 to 135.2)]. In contrast, Yemen [63.6 (95% UI: 51.9 to 76.4)], Afghanistan [69.3 (95% UI: 58.2 to 82.4)] and Sudan [69.7 (95% UI: 58.3 to 83.4)] had the three lowest rates (Fig. 1A and Table S2).

In 2019, the age-standardised death rates of PD varied from 3.5 to 11.1 cases per 100,000 population among the MENA countries. Also in 2019, the three countries with the highest estimated age-standardised death rates of PD were Qatar [11.1 (95% UI: 6.5 to 14.7)], Oman [9.1 (95% UI: 4.9 to 10.7)] and the United Arab Emirates [8.0 (95% UI: 4.8 to 10.4)]. In contrast, the lowest rates were found in Kuwait [3.5 (95% UI: 2.8 to 4.2)], Lebanon [4.4 (95% UI: 3.5 to 6.0)] and Tunisia [4.6 (95% UI: 3.4 to 6.0)] (Fig. 1B and Table S3).

In 2019, the national age-standardised DALY rates of PD ranged from 56.1 to 148.6 cases per 100,000 population. The highest DALY rates were observed in Qatar [148.6 (95% UI: 96.0 to 194.6)], the United Arab Emirates [130.5 (95% UI: 87.0 to 167.2)] and Oman [125.3 (95% UI: 79.0 to 144.5)]. Conversely, the lowest rates were seen in Kuwait [56.1 (95% UI: 47.2 to 66.0)], Lebanon [69.5 (95% UI: 57.3 to 91.6)] and Tunisia [72.7 (95% UI: 56.2 to 93.7)] (Fig. 1C and Table S4).

During the period 1990 to 2019, the age-standardised point prevalence increased in ten of the MENA countries, while no significant differences were observed in the remaining 11 countries. Oman [28.0% (95% UI: 14.0 to 44.8)], Saudi Arabia [27.7% (95% UI: 13.1 to 45.0)] and Turkey [22.3% (95% UI: 10.9 to 35.5)] showed the largest increases in the age-standardised point prevalence over the study period (Table S2 and Figure S1).

Only three countries in the MENA region showed an increase in the age-standardised death rates from 1990 to 2019, which were Morocco [46.6% (95% UI: 16.7 to 85.9)], Oman [44.7% (95% UI: 6.3 to 96.0)] and Qatar [35.3% (95% UI: 1.2 to 78.3)]. Kuwait [-25.1% (95% UI: -36.3 to -11.7)] was the only country to register a decrease during this period (Table S3 and Figure S2).

Morocco [39.6% (95% UI: 14.4 to 73.3)] was the only country to have a significant increase in the age-standardised DALY rates of PD, while Kuwait [-26.8% (95% UI: -36.5 to -14.7)] was the only country to have a significant decrease (Table S4 and Figure S3).

### Age and sex patterns

**Fig. 2:**
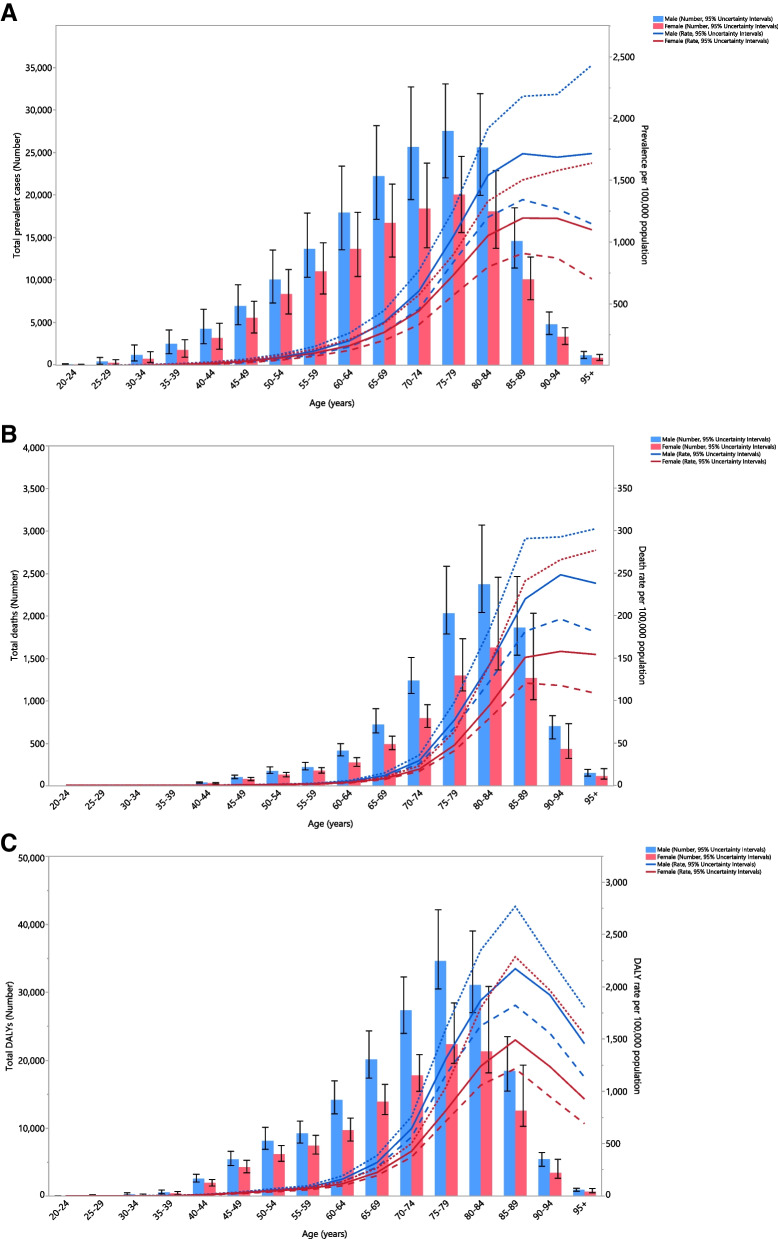
Number of prevalent cases and prevalence (A), number of deaths and death rate (B), and the number of DALYs and DALY rate (C) for Parkinson’s disease (per 100,000 population) in the Middle East and North Africa region, by age and sex in 2019; Dotted and dashed lines indicate 95% upper and lower uncertainty intervals, respectively. DALY = disability-adjusted-life-years. (Generated from data available from http://ghdx.healthdata.org/gbd-results-tool)

In 2019, the number of prevalent cases increased up to the 75–79 age group in both sexes, and then decreased to the oldest age group. The regional point prevalence of PD increased consistently with advancing age in both sexes, but a decrease was observed after the 90–94 age group in females (Fig. 2A). Furthermore, the number of deaths increased with increasing age up to the 80–84 age group, after which it decreased in both sexes. In addition, there was a clear increase in the regional death rate up to the 90–94 age group for both sexes, followed by a decrease for the remaining age groups (Fig. 2B). In 2019, the number of DALYs and the regional DALY rates rose with increasing age in both sexes up to the 75–79 (males) and 85–89 (females) age groups (Fig. 2C). Moreover, the number of prevalent cases, deaths and DALYs, along with their corresponding rates, were higher among males (than females) in all age groups.

**Fig. 3:**
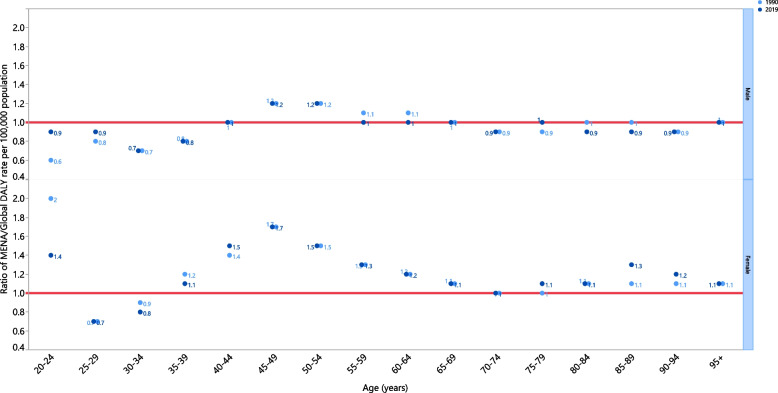
Ratio of the Middle East and North Africa region to the global Parkinson’s disease DALY rate according to age group and sex, 1990–2019. DALY = disability-adjusted-life-years. (Generated from data available from http://ghdx.healthdata.org/gbd-results-tool)

In 2019, males aged 20–39, 70–74, and 80–94 years, as well as females aged 25–34 and 75–95 + years old, had DALY rates that were lower than the global average (ratio of MENA/global DALY rate < 1). In addition, males aged 40–44, 55–69, 75–79, and 95^+^, as well as females aged 70–74 years, had DALY rates that were similar to their corresponding global rates (ratio of MENA/global DALY rate = 1). Moreover, males aged 45–54 years and females aged 20–24, 35–69, and older than 75 years old had DALY rates that were higher than the global average (ratio of MENA/global DALY rate > 1). The highest ratios of regional to global DALY rates in 2019 were found in males aged 45–54 years old (1.2) and females aged 45–49 (1.7). Compared with 1990, males aged 55–64 and 80–89 years old, and females aged 20–24 and 30–39 years old, had lower DALY rates in 2019. Furthermore, DALY rates increased from 1990 to 2019 for the 20–29 age group among males and the 40–44, 75–79, and 85–94 age groups among females (Fig. 3).

### Association with the socio-demographic index (SDI)

**Fig. 4:**
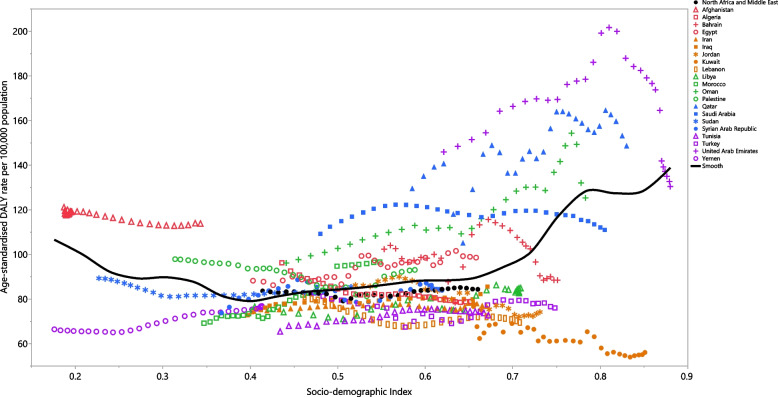
Age-standardised DALY rates of Parkinson’s disease for the 21 countries and territories in 2019, by SDI; Expected values based on the Socio-demographic Index and disease rates in all locations are showns as the black line. Each point shows the observed age-standardised DALY rate for each country in 2019. DALY = disability-adjusted-life-years. SDI = Socio-demographic Index (Generated from data available from http://ghdx.healthdata.org/gbd-results-tool)

Across the period 1990 to 2019, the burden of PD decreased with increasing socio-economic development, up to an SDI of around 0.4 and then the burden of the disease gradually increased with higher SDI levels. SDI levels higher than 0.65 were associated with significant increases in the burden of PD. In several countries (e.g., Afghanistan, Egypt and Qatar), the burden of PD was higher than expected, while in others (e.g., Iran, Iraq, Kuwait, Lebanon, Libya, Tunisia, Turkey and Yemen), the burden was lower than expected (Fig. 4).

## Discussion

The current study provided an overview of the most comprehensive and up-to-date data concerning the burden of PD in the MENA region over the past 30 years. Our findings highlighted a pronounced upward trend in the age-standardised point prevalence of PD from 1990 to 2019. Nonetheless, we found that the death and DALY rates did not change substantially over the last three decades. Furthermore, the point prevalence, death and DALY rates were higher among males than females of all ages and peaked among older adults. Comparing the attributable burden of PD in MENA to the corresponding burden at the global level, in 2019 females showed higher DALY rates while males had lower rates in nearly all age groups. Moreover, a slight increase in the burden of PD was found with increasing SDI levels.

While there was no previous study exclusively targeting the burden of PD at the regional level, several investigations have reported the attributable burden at the global level [8, 9]. In 2016, PD was ranked 12^th^ among the neurological disorders in the MENA region, in terms of the age-standardised DALY rate [13]. Ou and colleagues utilised GBD 2019 data to illustrate the burden and trends associated with PD around the world [9]. They found that the age-standardised point prevalence of PD was 106.3 per 100,000 population in 2019, and that this rate had increased by 155.5% since 1990 [9]. The results of the present study showed a more modest increase in the MENA region, where the age-standardised point prevalence was 82.6 per 100,000 population, which was 15.4% higher than in 1990. Furthermore, in line with the results of the current study, previous research has shown that the prevalence, death, and DALYs due to PD peaked among older adults and that males had higher rates in almost all age groups [8, 9].

The rising prevalence of PD in the MENA region might be due to population aging, together with changes in the environment, such as increasing air pollution and alterations in dietary patterns and lifestyles. Since 1990, the population aged 80 years or older has approximately doubled in MENA [14]. According to the GBD 2017 findings, it has been estimated that more than half (51.3% [48.0–54.8]) of the burden was attributable to aging-related diseases in the MENA region [15]. Therefore, it could be assumed that the elevated prevalence of PD among people in this region might be partly due to an increase in the numbers of older people and a longer disease duration. As reported by the GBD 2016 study, advancing age has resulted in an approximately 22% increase in the age-standardised prevalence of PD since 1990 [8]. Furthermore, several reports have concluded that long-term exposure to ambient particulate matter can increase the risk of developing PD later in life [16–18]. A recent systematic review and meta-analysis of 80 publications demonstrated that exposure to PM_2.5_ was associated with a 34% increase in the long-term incidence of PD (OR 1.34, 95%CI 1.04–1.73) [19]. Furthermore, a 2013 study using GBD data revealed that the MENA region had the highest concentration of PM_2.5_ in the world, and that this was due to windblown mineral dust as a result of rapid industrialisation [20]. Moreover, research has shown that a healthy diet filled with vegetables and the absence of processed foods can prevent of the onset of PD [21]. Malnutrition, a diet state where essential nutrients are missing, has been shown to increase the risk of PD [22]. According to the GBD 2019 risk factor study, malnutrition was the third leading risk factor for attributable DALYs in the MENA region. Furthermore, psychiatric disorders may also increase the risk of PD [23], with the latest GBD iteration showing that in MENA the prevalence rates for most mental disorders were higher than the global average [24]. Consequently, the high prevalence of PD in this region needs to be addressed and effective measures are required to alleviate the burden of related risk factors.

Our findings demonstrated that the point prevalence, death and DALY rates attributable to PD were substantially higher among males than females. Similarly, a previous paper aimed at measuring the burden and trends of neurological disorders, using GBD 2016 data, found that the male to female ratio for the prevalence, death, and DALYs due to PD were 1.4, 1.8, and 1.7, respectively [13]. The gender differences may be due to gender differences in the frequency of exposure to the different risk factors and perhaps an interaction with sex-related differences. For instance, it has been reported that among male subjects, a lack of coffee consumption, head trauma, and pesticide exposure were independent risk factors and cases who had at least one of these risk factors had an approximately five-time higher risk of developing PD (OR 5.28; 95%CI 2.67–10.43). Among females, those who had either anemia or a higher level of education were found to become more vulnerable to the development of PD (OR 4.71; 95%CI 1.61–13.72) [25]. Moreover, whether estrogen has a neuroprotective role has long been debated, and this might partly justify the lower prevalence of PD among females [26, 27].

Traumatic brain injury is a well-known risk factor for the development of PD at an older age [28]. In 2016, the highest prevalence rates of traumatic brain injury were found in Qatar and third highest was in the United Arab Emirates [29]. In parallel to our results, Qatar and the United Arab Emirates had two of the highest age-standardised prevalence rates attributable to PD. Thus, effective management of the burden attributable to PD necessitates the implementation of interventions to lower the rates of falls and motor vehicle injuries. Nevertheless, several investigations have highlighted the protective role smoking has in the subsequent onset of PD [30–32]. A previous meta-analysis of 69 publications found an inverse relationship between smoking and the long-term incidence of PD [33]. Among MENA countries, Oman and Turkey had the largest decreases in the point prevalence of smoking since 1990 [34]. In support of the previous meta-analysis, our results showed that Oman and Turkey had the largest increases in the prevalence of PD over the period 1990 to 2019. This finding does not imply that smoking is recommended to reduce the risk of PD, since many epidemiological studies have found that smoking is a leading risk factor for numerous other non-communicable diseases. Nevertheless, developing novel treatments using chemicals derived from tobacco may help to reduce the risk for individuals at a higher risk of PD or to reduce the risk of disease progression for those already affected.

Considering PD as an age-dependent disorder, the demographic metrics of a population clearly influence the number of affected patients. For instance, whenever the net reproductive rate increases and the life expectancy decreases in a targeted community, we would expect the rate of individuals developing PD to decline, due to the transition towards a younger population structure. In other words, fewer individuals would reach the prevalent age range for PD. Within the MENA region in 2019, Afghanistan and Yemen had the lowest life expectancies of 63.3 and 67.7 years old, respectively [35]. In addition, the net reproductive rates were also the highest in these two nations [35]. In agreement with our results, the lowest frequency of PD cases were found in Afghanistan and Yemen, consistent with their youthful populations and low life expectancies.

When we compared the regional DALYs to the average global estimates, MENA had higher DALYs in almost all age groups. This might imply that the affected patients are not being fully supported by the healthcare services to help reduce complications later in the course of the disease. Therefore, improving resources that meet the needs of the growing numbers of the elderly with PD and planning for accessible rehabilitation programs could help offer a satisfactory quality of life for these patients.

Examining the relationship that SDI has with the burden of PD, we found a slight positive association between the age-standardised DALY rates and the developmental status of the MENA countries. In contrast, the GBD 2016 study found a steep increase in the age-standardised DALY rates with higher SDI levels across the world [8]. Furthermore, the 2019 global results showed no association between the attributable burden of PD and SDI [9]. This might imply that the burden of PD has been moderated among less developed and more developed countries in recent years. A similar pattern of findings could be generalised to the MENA region, in which the developed countries might have contributed to a larger portion of the disease burden in recent years. Furthermore, the increased burden of PD among the developed countries of MENA is likely linked to the improved therapeutic options available that prolong the duration of the disease. Thus, the remarkable increase in the disease burden may also be associated with increased disabilities, due to the extended life expectancy and higher prevalence of long-term psychiatric complications, such as psychosis, depression, and anxiety [36]. Furthermore, people in developed countries are probably exposed to higher levels of the risk factors associated with industrialisation, such as pesticides [37], solvents [38], heavy metals [39], and pollutants which might increase the incidence of PD and subsequently result in a higher disease burden among more industrialised countries. Moreover, SDI can be used as an indicator of educational attainment. It has been reported that better educational levels were linked with a higher risk of PD [40], which may be another reason for the higher burden of PD in developed countries. Consequently, if the reported 15% increase in the point prevalence of PD across MENA is confirmed by further high-quality investigations, we can expect that the burden will continue to rise for many years to come. In addition to the ongoing conservative increase in the prevalence of PD, if the population continues to age, clinical management keeps improving survival rates, and if environmental risk factors stay steady or grow, then we can anticipate an even larger rise in the prevalence of the disease in the near future [41, 42]. Moreover, with a large reduction in the region’s smoking rate over the last few years [34], further increases in the number of PD cases are even more likely [43]. Due to the current lack of appropriate strategies and the absence of suitably equipped infrastructure in most MENA countries, we would anticipate an escalated burden from PD in the near future. Thus, there is an urgent need for research to identify interventions for the effective prevention and treatment of PD [44].

### Strengths and limitations of the study

This is the first study to evaluate the attributable burden of PD by age, sex, and SDI in the MENA region, over the period 1990 to 2019. Nevertheless, we acknowledge that the present research has several shortcomings that must be considered when interpreting our findings. Firstly, there were few high-quality data sources in several MENA countries, and the extensive and complicated assumptions made in the GBD project might reduce the validity and reliability of our findings. Therefore, more high-quality epidemiological studies are needed, particularly in the low-income countries, to understand the trends and to guide policymakers in planning widespread interventions. Secondly, PD is a heterogeneous disorder, which presents with different phenotypes, progression patterns, and accompanying cognitive impairments [45]. As a result, the misdiagnosis of PD occurs routinely among clinicians, with trained specialist physicians being required to make an accurate diagnosis of PD. Thus, this might have affected the robustness and certainty of our results. Thirdly, due to the limitations of the GBD study, we could not identify the impact of the different risk factors in the development of PD. This highlights the need for future iterations of the GBD project to include risk factors. Fourthly, we did not report the neurobiological connections in relation to the development of PD, which should be considered in future research.

## Conclusions

A pronounced upward trend was observed in the point prevalence of PD over the last three decades in MENA, indicating that the disease will continue to be a growing challenge for public health. Considering the rapid growth of longevity in this region, it is likely that the prevalence of PD will continue to rise in the coming years. Moreover, although the burden of PD did not greatly change from 1990 to 2019, the burden could be efficiently decreased in MENA by planning and implementing programs to support the elderly with PD, improving regional access to care facilities and effective treatments, allocating more resources for research in order to understand the underlying risk factors, and via the development of novel therapies.

## Supplementary Information

**Additional file 1:** **Supplementary fig 1.** 

**Additional file 2:** **Supplementary fig 2.**

**Additional file 3:** **Supplementary fig 3.** 

**Additional file 4:** **Supplementary table 1.**

**Additional file 5:** **Supplementary table 2.**

**Additional file 6:** **Supplementary table 3.**

**Additional file 7:** **Supplementary table 4.**

## Data Availability

The data used for these analyses are all publicly available at http://ghdx.healthdata.org/gbd-results-tool.
