# Supplementary material for: The burden of Parkinson’s disease in the Middle East and North Africa region, 1990–2019: results from the global burden of disease study 2019
Source: BMC Public Health. 2023 Jan 16;23:107. doi: 10.1186/s12889-023-15018-x (PMC9841703; doi:10.1186/s12889-023-15018-x)
Supplement: Supplementary file 3 — Additional file 3: Supplementary fig 3. [file 12889_2023_15018_MOESM3_ESM.docx]

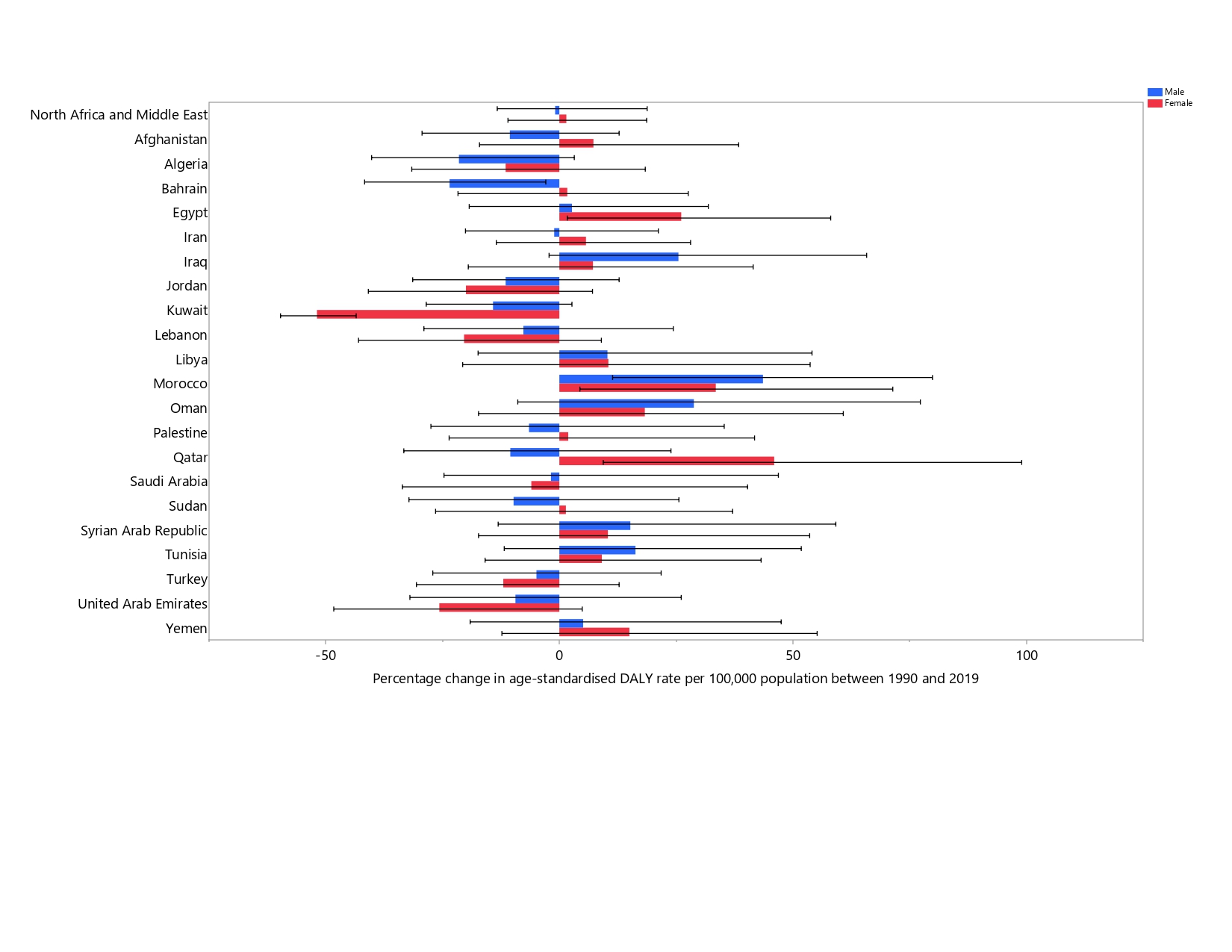


**Figure S3:** The percentage change in the age-standardised DALY rate of Parkinson’s disease in the Middle East and North Africa region from 1990 to 2019, by sex and country. DALY= disability-adjusted-life-years. (Generated from data available from <http://ghdx.healthdata.org/gbd-results-tool>).
