# Supplementary material for: The burden of Parkinson’s disease in the Middle East and North Africa region, 1990–2019: results from the global burden of disease study 2019
Source: BMC Public Health. 2023 Jan 16;23:107. doi: 10.1186/s12889-023-15018-x (PMC9841703; doi:10.1186/s12889-023-15018-x)
Supplement: Supplementary file 4 — Additional file 4: Supplementary table 1. [file 12889_2023_15018_MOESM4_ESM.doc]

| **Table S1: Sequelae for Parkinson’s disease and the corresponding disability weights in the Global Burden of Disease 2019 Study** | | |
| --- | --- | --- |
| **Sequelae** | **Lay description** | **Disability weights (95% CI)** |
| Mild | Has mild tremors and moves a little slowly, but is able to walk and do daily activities without assistance. | 0.01  (0.005–0.019) |
| Moderate | Has moderate tremors and moves slowly, which causes some difficulty in walking and daily activities. The person has some trouble swallowing, talking, sleeping, and remembering things. | 0.267  (0.181–0.372) |
| Severe | Has severe tremors and moves very slowly, which causes great difficulty in walking and daily activities. The person falls easily and has a lot of difficulty talking, swallowing, sleeping, and remembering things. | 0.575  (0.396–0.73) |
