# Supplementary material for: The burden of Parkinson’s disease in the Middle East and North Africa region, 1990–2019: results from the global burden of disease study 2019
Source: BMC Public Health. 2023 Jan 16;23:107. doi: 10.1186/s12889-023-15018-x (PMC9841703; doi:10.1186/s12889-023-15018-x)
Supplement: Supplementary file 5 — Additional file 5: Supplementary table 2. [file 12889_2023_15018_MOESM5_ESM.docx]

| **Table S2: Prevalence of Parkinson’s disease in 1990 and 2019 and the percentage change in the age-standardised rates per 100,000 in the Middle East North and Africa region** | | | | | |
| --- | --- | --- | --- | --- | --- |
|  | **1990** | | **2019** | | **PCs in ASRs per 100,000** |
|  | **No (95% UI)** | **ASRs per 100,000 (95% UI)** | **No (95% UI)** | **ASRs per 100,000 (95% UI)** |  |
| **North Africa and Middle East** | **103476 (87446 , 122084)** | **71.6 (60.3 , 84)** | **309887 (264964 , 362774)** | **82.6 (70.2 , 95.6)** | **15.4 (11.5 , 20)** |
| **Afghanistan** | **4544 (3675 , 5600)** | **71.7 (57.9 , 87.5)** | **7352 (6145 , 8807)** | **69.3 (58.2 , 82.4)** | **-3.3 (-12.9 , 8)** |
| **Algeria** | **7547 (6249 , 9108)** | **72.9 (60.9 , 87.2)** | **23956 (19889 , 28839)** | **80.4 (66.5 , 96.8)** | **10.2 (-1.1 , 22)** |
| **Bahrain** | **114 (93 , 138)** | **83.5 (67.9 , 99.8)** | **674 (552 , 825)** | **94 (75.9 , 113.4)** | **12.5 (-5.2 , 33.3)** |
| **Egypt** | **17622 (14406 , 21123)** | **71.9 (58.7 , 85.9)** | **43837 (36133 , 52603)** | **84.4 (69.1 , 101)** | **17.3 (3.6 , 30.7)** |
| **Iran (Islamic Republic of)** | **15661 (13068 , 18696)** | **75.3 (62.6 , 89.6)** | **56514 (47335 , 66920)** | **85.1 (70.2 , 101.3)** | **13.1 (10.4 , 15.8)** |
| **Iraq** | **4901 (4063 , 5859)** | **70.6 (58.5 , 84.7)** | **14382 (11802 , 17136)** | **74.2 (61.1 , 87.9)** | **5.1 (-4.3 , 17.1)** |
| **Jordan** | **785 (657 , 933)** | **74.7 (62.2 , 87.3)** | **4127 (3383 , 4884)** | **77 (63.4 , 91.5)** | **3 (-11.5 , 16.6)** |
| **Kuwait** | **381 (311 , 460)** | **78 (64.1 , 93.8)** | **1581 (1292 , 1923)** | **71.4 (59.1 , 86.1)** | **-8.5 (-23.9 , 9.9)** |
| **Lebanon** | **1391 (1153 , 1645)** | **71 (58.8 , 83.3)** | **4065 (3476 , 4757)** | **77.6 (66.5 , 90.9)** | **9.3 (-3.1 , 23.6)** |
| **Libya** | **1222 (1019 , 1447)** | **74.1 (61.5 , 87.4)** | **3830 (3183 , 4453)** | **84.1 (69.3 , 98.3)** | **13.5 (2.3 , 27.9)** |
| **Morocco** | **7322 (5956 , 8753)** | **61 (49.7 , 72.6)** | **20470 (17155 , 23848)** | **73.7 (61.9 , 85.8)** | **20.7 (6.3 , 39)** |
| **Oman** | **435 (358 , 524)** | **87.8 (71.9 , 105.2)** | **1362 (1125 , 1661)** | **112.5 (92.9 , 135.2)** | **28 (14 , 44.8)** |
| **Palestine** | **587 (483 , 698)** | **76.7 (62.8 , 91.2)** | **1517 (1259 , 1792)** | **78.3 (65.2 , 92.4)** | **2.1 (-8.8 , 14.4)** |
| **Qatar** | **82 (67 , 101)** | **99.2 (81.9 , 118.5)** | **750 (594 , 951)** | **119.3 (98.3 , 142.9)** | **20.2 (6.9 , 36.3)** |
| **Saudi Arabia** | **3981 (3290 , 4710)** | **84.3 (68.7 , 101.1)** | **13291 (11019 , 15910)** | **107.6 (90.4 , 127.8)** | **27.7 (13.1 , 45)** |
| **Sudan** | **5632 (4584 , 6686)** | **68.2 (56 , 80.9)** | **11401 (9527 , 13651)** | **69.7 (58.3 , 83.4)** | **2.1 (-9.9 , 16.1)** |
| **Syrian Arab Republic** | **3206 (2694 , 3805)** | **73.1 (61.2 , 85.8)** | **8349 (6932 , 9929)** | **81.6 (67.8 , 97)** | **11.6 (-1.3 , 26.8)** |
| **Tunisia** | **3019 (2508 , 3600)** | **69.2 (57.3 , 82.1)** | **9312 (7834 , 10877)** | **79.6 (66.9 , 93.2)** | **15.1 (1.8 , 30.4)** |
| **Turkey** | **22297 (18128 , 26774)** | **71.1 (57.5 , 85.6)** | **72458 (59364 , 86774)** | **86.9 (71 , 104.1)** | **22.3 (10.9 , 35.5)** |
| **United Arab Emirates** | **313 (255 , 382)** | **103.8 (85.2 , 124.5)** | **3131 (2506 , 3880)** | **116 (97.6 , 138.8)** | **11.8 (0.6 , 23.6)** |
| **Yemen** | **2365 (1911 , 2854)** | **58 (47 , 69.6)** | **7214 (5874 , 8683)** | **63.6 (51.9 , 76.4)** | **9.7 (-1.8 , 24.7)** |
| **Abbreviations:** ASRs: Age-standardised rates; PCs: Percentage changes; UI: Uncertainty interval. Generated from data available from <http://ghdx.healthdata.org/gbd-results-tool> | | | | | |
