# Supplementary material for: The burden of Parkinson’s disease in the Middle East and North Africa region, 1990–2019: results from the global burden of disease study 2019
Source: BMC Public Health. 2023 Jan 16;23:107. doi: 10.1186/s12889-023-15018-x (PMC9841703; doi:10.1186/s12889-023-15018-x)
Supplement: Supplementary file 6 — Additional file 6: Supplementary table 3. [file 12889_2023_15018_MOESM6_ESM.docx]

| **Table S3: Deaths due to Parkinson’s disease in 1990 and 2019 and the percentage change in the age-standardised rates per 100,000 in the Middle East and North Africa region** | | | | | |
| --- | --- | --- | --- | --- | --- |
|  | **1990** | | **2019** | | **PCs in ASRs per 100,000** |
|  | **No (95% UI)** | **ASRs per 100,000 (95% UI)** | **No (95% UI)** | **ASRs per 100,000 (95% UI)** |  |
| **North Africa and Middle East** | **6006 (5245 , 7808)** | **5.2 (4.5 , 6.9)** | **16784 (14607 , 21649)** | **5.3 (4.6 , 6.9)** | **2.3 (-10 , 19)** |
| **Afghanistan** | **371 (280 , 518)** | **6.9 (5.4 , 9.6)** | **560 (431 , 715)** | **6.8 (5.3 , 8.5)** | **-2.3 (-22.7 , 20.3)** |
| **Algeria** | **439 (349 , 554)** | **6.7 (5.3 , 8.5)** | **1283 (1019 , 1583)** | **5.4 (4.4 , 6.7)** | **-18.7 (-36.3 , 4.6)** |
| **Bahrain** | **6 (5 , 6)** | **6.5 (5.7 , 7.3)** | **21 (15 , 27)** | **6 (4.2 , 7.4)** | **-7.7 (-29.9 , 17.9)** |
| **Egypt** | **1045 (939 , 1581)** | **5.4 (4.8 , 8.4)** | **2439 (1842 , 3675)** | **6 (4.6 , 9.3)** | **12.2 (-10.6 , 38.2)** |
| **Iran (Islamic Republic of)** | **643 (554 , 724)** | **4.5 (3.8 , 5.3)** | **2848 (2432 , 3155)** | **4.8 (4.1 , 5.3)** | **6.7 (-12.6 , 28.4)** |
| **Iraq** | **279 (217 , 418)** | **4.6 (3.6 , 6.7)** | **842 (684 , 1150)** | **5.7 (4.6 , 7.4)** | **22.8 (-3.6 , 58)** |
| **Jordan** | **44 (36 , 56)** | **5.7 (4.6 , 7)** | **192 (160 , 228)** | **4.9 (4.1 , 5.8)** | **-13.2 (-30 , 8.6)** |
| **Kuwait** | **16 (14 , 18)** | **4.7 (4 , 5.2)** | **58 (47 , 69)** | **3.5 (2.8 , 4.2)** | **-25.1 (-36.3 , -11.7)** |
| **Lebanon** | **83 (70 , 103)** | **5.1 (4.2 , 6.2)** | **226 (180 , 312)** | **4.4 (3.5 , 6)** | **-14.1 (-37.2 , 15.7)** |
| **Libya** | **67 (53 , 83)** | **4.6 (3.6 , 5.6)** | **203 (150 , 265)** | **5 (3.7 , 6.5)** | **9.9 (-18.1 , 50.5)** |
| **Morocco** | **404 (318 , 516)** | **4.1 (3.2 , 5.3)** | **1390 (1122 , 1614)** | **6 (4.9 , 7)** | **46.6 (16.7 , 85.9)** |
| **Oman** | **21 (13 , 27)** | **6.3 (3.6 , 8)** | **60 (37 , 70)** | **9.1 (4.9 , 10.7)** | **44.7 (6.3 , 96)** |
| **Palestine** | **42 (28 , 55)** | **6.4 (4.3 , 8.3)** | **92 (61 , 107)** | **6.2 (4 , 7.2)** | **-2.5 (-26.2 , 36.2)** |
| **Qatar** | **3 (2 , 4)** | **8.2 (5.1 , 10.6)** | **19 (13 , 26)** | **11.1 (6.5 , 14.7)** | **35.3 (1.2 , 78.3)** |
| **Saudi Arabia** | **270 (187 , 333)** | **7.3 (4.9 , 9)** | **562 (414 , 665)** | **7.3 (5 , 8.7)** | **0.2 (-22.2 , 47.7)** |
| **Sudan** | **365 (262 , 502)** | **5.2 (3.8 , 7.1)** | **710 (557 , 898)** | **5.2 (4.1 , 6.5)** | **0.1 (-24.7 , 36.8)** |
| **Syrian Arab Republic** | **173 (119 , 211)** | **4.7 (3.2 , 5.7)** | **434 (286 , 555)** | **5.7 (3.7 , 7.2)** | **21.1 (-8.4 , 61.8)** |
| **Tunisia** | **144 (120 , 176)** | **4.1 (3.4 , 5)** | **485 (356 , 646)** | **4.6 (3.4 , 6)** | **12 (-16.4 , 45.8)** |
| **Turkey** | **1453 (1184 , 2478)** | **5.2 (4.2 , 8.9)** | **3838 (2916 , 7139)** | **4.9 (3.7 , 9.1)** | **-6.8 (-27 , 16.1)** |
| **United Arab Emirates** | **14 (7 , 19)** | **9.1 (4.2 , 12)** | **90 (60 , 123)** | **8 (4.8 , 10.4)** | **-12.8 (-32.3 , 23.5)** |
| **Yemen** | **119 (92 , 162)** | **3.8 (2.9 , 5.1)** | **413 (322 , 540)** | **4.6 (3.6 , 6)** | **21.9 (-3.2 , 58.7)** |
| **Abbreviations:** ASRs: Age-standardised rates; PCs: Percentage changes; UI: Uncertainty interval. Generated from data available from <http://ghdx.healthdata.org/gbd-results-tool> | | | | | |
