# Supplementary material for: The burden of Parkinson’s disease in the Middle East and North Africa region, 1990–2019: results from the global burden of disease study 2019
Source: BMC Public Health. 2023 Jan 16;23:107. doi: 10.1186/s12889-023-15018-x (PMC9841703; doi:10.1186/s12889-023-15018-x)
Supplement: Supplementary file 7 — Additional file 7: Supplementary table 4. [file 12889_2023_15018_MOESM7_ESM.docx]

| **Table S4: DALYs due to Parkinson’s disease in 1990 and 2019 and the percentage change in the age-standardised rates per 100,000 in the Middle East and North Africa region** | | | | | |
| --- | --- | --- | --- | --- | --- |
|  | **1990** | | **2019** | | **PCs in ASRs per 100,000** |
|  | **No (95% UI)** | **ASRs per 100,000 (95% UI)** | **No (95% UI)** | **ASRs per 100,000 (95% UI)** |  |
| **North Africa and Middle East** | **113988 (99982 , 140731)** | **83.6 (73.3 , 104.6)** | **300698 (266277 , 365360)** | **84.4 (74.7 , 103.2)** | **0.9 (-10.2 , 15.7)** |
| **Afghanistan** | **7589 (5640 , 10454)** | **121.1 (93 , 166)** | **11574 (8845 , 14866)** | **113.8 (88.7 , 143.3)** | **-6 (-25.7 , 16.3)** |
| **Algeria** | **8306 (6721 , 10141)** | **96.3 (78.8 , 118.8)** | **21708 (17579 , 26334)** | **78.9 (64.1 , 95.1)** | **-18.1 (-35.7 , 3.6)** |
| **Bahrain** | **117 (100 , 133)** | **102.3 (89.4 , 115)** | **467 (340 , 573)** | **88.5 (64.1 , 106.6)** | **-13.5 (-32.1 , 7.5)** |
| **Egypt** | **20554 (18656 , 28621)** | **88.2 (79.7 , 125.4)** | **48343 (37833 , 67875)** | **98.6 (77.1 , 142.4)** | **11.7 (-9.4 , 36.5)** |
| **Iran (Islamic Republic of)** | **13806 (12015 , 15388)** | **74.5 (64.7 , 83.5)** | **48779 (42378 , 53932)** | **76.8 (66.9 , 84.9)** | **3.1 (-12.9 , 20.5)** |
| **Iraq** | **4843 (3857 , 7098)** | **72.8 (58 , 106.8)** | **14973 (12204 , 20254)** | **85.6 (71 , 115.2)** | **17.5 (-5.7 , 47.1)** |
| **Jordan** | **811 (684 , 1006)** | **86.3 (71.3 , 106.5)** | **3534 (3007 , 4138)** | **74.2 (63 , 86.7)** | **-14 (-29.8 , 5)** |
| **Kuwait** | **328 (292 , 365)** | **76.7 (68.2 , 85.3)** | **1052 (894 , 1255)** | **56.1 (47.2 , 66)** | **-26.8 (-36.5 , -14.7)** |
| **Lebanon** | **1513 (1289 , 1794)** | **81.1 (69.1 , 96.7)** | **3662 (3016 , 4825)** | **69.5 (57.3 , 91.6)** | **-14.2 (-35.2 , 11.2)** |
| **Libya** | **1210 (988 , 1447)** | **76.5 (62.7 , 92.2)** | **3712 (2836 , 4719)** | **84.8 (65.3 , 108.4)** | **10.9 (-15.2 , 45.8)** |
| **Morocco** | **7868 (6259 , 9629)** | **69.2 (55.4 , 85.1)** | **25269 (20568 , 29607)** | **96.6 (79.3 , 111.9)** | **39.6 (14.4 , 73.3)** |
| **Oman** | **409 (293 , 514)** | **96.2 (64.6 , 121.5)** | **1097 (785 , 1265)** | **125.3 (79 , 144.5)** | **30.3 (-2.2 , 70.4)** |
| **Palestine** | **709 (503 , 910)** | **97.9 (69.2 , 125)** | **1614 (1142 , 1855)** | **93.1 (64.9 , 106.8)** | **-4.9 (-26.4 , 29.1)** |
| **Qatar** | **79 (59 , 100)** | **129.5 (89.4 , 165)** | **488 (360 , 643)** | **148.6 (96 , 194.6)** | **14.8 (-11.6 , 49.8)** |
| **Saudi Arabia** | **4714 (3388 , 5790)** | **109.3 (78.6 , 132.5)** | **11587 (9409 , 13541)** | **111 (83.8 , 129.8)** | **1.6 (-19.9 , 43.5)** |
| **Sudan** | **7279 (5290 , 9878)** | **89.3 (66 , 119.2)** | **13238 (10419 , 16768)** | **85.5 (67.8 , 107.5)** | **-4.3 (-26.5 , 26.1)** |
| **Syrian Arab Republic** | **3024 (2223 , 3650)** | **73.8 (53.5 , 88.3)** | **7787 (5490 , 9816)** | **83.9 (58.4 , 104.9)** | **13.7 (-12.2 , 49.7)** |
| **Tunisia** | **2705 (2323 , 3227)** | **65.4 (56.2 , 77.8)** | **8271 (6418 , 10676)** | **72.7 (56.2 , 93.7)** | **11.1 (-13.8 , 41)** |
| **Turkey** | **25115 (20956 , 39914)** | **82.8 (69 , 133)** | **62178 (48654 , 105236)** | **76.1 (59.4 , 129.7)** | **-8.2 (-25.9 , 11.9)** |
| **United Arab Emirates** | **355 (219 , 459)** | **145.9 (78 , 185.9)** | **2896 (2100 , 3866)** | **130.5 (87 , 167.2)** | **-10.6 (-29.6 , 21.4)** |
| **Yemen** | **2578 (1998 , 3493)** | **66.5 (52.1 , 89.1)** | **8163 (6383 , 10687)** | **76.8 (60.8 , 98.7)** | **15.5 (-7.8 , 49.7)** |
| **Abbreviations:** DALY: disability-adjusted life-year, ASRs: Age-standardised rates; PCs: Percentage changes; UI: Uncertainty interval. Generated from data available from <http://ghdx.healthdata.org/gbd-results-tool> | | | | | |
